# High IFN-gamma and TNF production by peripheral NK cells of Colombian patients with different clinical presentation of *Plasmodium falciparum*

**DOI:** 10.1186/1475-2875-11-38

**Published:** 2012-02-08

**Authors:** Olga Agudelo, Julio Bueno, Andres Villa, Amanda Maestre

**Affiliations:** 1 Grupo Salud y Comunidad, Facultad de Medicina, Universidad de Antioquia, Medellin, Colombia; 2 Grupo Reproducción, Facultad de Medicina, Universidad de Antioquia, Medellin, Colombia; 3 Escuela de Microbiología, Universidad de Antioquia, Medellin, Colombia

## Abstract

**Background:**

In Colombia*, Plasmodium falciparum *infection rarely results in severe disease or mortality compared to infections in African populations. During natural infection NK cells exhibit a cytolytic effect and regulate dendritic cells, macrophages, neutrophils as well as affect antigen specific T and B cell responses. To characterize the NK cells in *P. falciparum *infected patients of a highly endemic region of Colombia, the degree of NK proliferation and production of IFN gamma and TNF production in these cells were explored.

**Methods:**

Seventeen patients with acute and three with severe *P. falciparum *malaria patients from the Northwest region of the country were recruited in the study. In addition, 20 healthy controls were included: 10 from Medellin (no-transmission area) and 10 from the Uraba region (a malaria endemic area). Immunophenotypic analysis of peripheral mononuclear cells was performed by FACS to detect total number of NK cells, subtypes and intracellular IFNγ and TNF production by NK cells in the different patient groups.

**Results:**

The total mean CD56^+^/CD3^- ^NK cell proportions in acute and severe malaria subjects were 9.14% (7.15%CD56^dim^, 2.01%CD56^bright^) and 19.62% (16.05%CD56^dim^, 3.58%CD56^bright^), respectively, in contrast to healthy controls from endemic (total mean CD56^+^/CD3^-^1.2%) and non-endemic area (total mean CD56^+^/CD3^- ^0.67%). Analysis of basal IFNγ and TNF levels confirmed the CD56^bright ^NK population as the main cytokine producer (*p *< 0.0001) in the groups affected with malaria, with the CD56^dim ^NK cell exhibiting the highest potential of TNF production after stimulus in the acute malaria group.

**Conclusions:**

The results confirm the important role of not only CD56^bright ^but also of CD56^dim ^NK cell populations as producers of the two cytokines in malaria patients in Colombia.

## Background

The clinical presentation of malaria depends on the confluence of diverse factors, including the degree of natural and acquired specific immunity, host's genetic composition, age, occupation and social and economic factors of the population [1].

Malaria in Colombia is highly endemic in the north-west, Pacific Coast and Amazon regions, all among the most deprived of the country due to social-political conflicts resulting in migrations and poverty. Previous studies in the northwest of Colombia, confirmed that children below 12 years of age are highly susceptible to malaria with a mean seven years of age for clinical presentation with malaria [2]. About 70% of this young population was affected by chronic malnutrition and 85% with intestinal parasitism, two conditions with important effects on the immune fitness of malaria affected individuals [2,3].

Despite the high frequency of *Plasmodium falciparum *infection, severe or fatal malaria cases are rare in the country. Out of the 79,909 malaria cases (72% *Plasmodium vivax*-27% *P. falciparum*) reported in 2009, 307 were severe (1.4% of *P. falciparum *cases) and the fatality rate was 0.04%[4]. This is in striking contrast to reports from African populations, where around 0.4% mortality rates were reported in the same year, most of them in children under 5 years of age [5]. In Colombia, is the 20-24 age group the most frequently affected by malaria, with around 15% of total cases, followed by the 15-19 age group (around 14%) and the 10-14 age group (around 12%). For severe malaria, the most commonly affected groups is the 20-24 age group (around 21%) and the 15-19 age group (around 13%)[6]. This is evidence of a clear-cut difference in the age pattern of severe malaria presentation between Colombia and African countries.

For many years, the importance of effective acquired immune response to protect against severe *P. falciparum *infection has been known. In this sense, both innate and adaptative immune responses, constitute a key component in subsequent *Plasmodium *challenges by reducing parasitaemia during the acute phase of the disease [7]. After infection with a microorganism, natural killer (NK) cells lymphocyte lineage cells exhibit a cytolytic effect, which, can directly induce the death of infected cells in absence of specific immunization. Subsequently, NK cells have been recognized as major producers of interferon-γ (IFN-γ) and other cytokines, either pro-inflammatory or anti-inflammatory, including tumor necrosis factor (TNF), interleukin (IL)-10, and growth factors such as GM-CSF (granulocyte macrophage colony-stimulating factor), G-CSF (granulocyte colony stimulating factor), and IL-3. NK cells also secrete many chemokines, including CCL2 (MCP-1), CCL3 (MIP1-α), CCL4 (MIP1-β), CCL5 (RANTES), XCL1 (lymphotactin), and CXCL8 (IL-8) [8].

The evidence gathered so far confirms that NK cells can positively [9,10] or negatively [11] influence the host's T and B cell immunity, depending on the nature of the antigenic challenge. Therefore, in addition to their cytolytic effect, NK cells can also regulate dendritic cells, macrophages, neutrophils [12] and affect antigen specific T and B cell responses [13]. According to the expression density of CD56, NK cells can be divided into CD56^dim ^representing the vast majority of human NK cells and a small distinct population of CD56^bright ^NK cells [14,15]. Almost all CD56^bright ^NK cells fail to express CD16 and exhibit a weak cytotoxic activity. In healthy humans, CD56^bright ^cells comprise up to 10% of all NK cells. Granules of CD56^dim ^cells are richer in perforin and granzyme A, which results in a more effective cytolysis compared to CD56^bright ^NK cells. However, CD56^bright ^NK cells are more efficient in producing the pro-inflammatory cytokines IFNγ and TNF [16].

The presence of severe malaria has been strongly attributed to an exaggerated immune response of the host towards parasite antigens and several authors reported on the positive correlation between high TNF production and cerebral malaria in humans as well as in animal models [17]. Parasite genetic diversity does not seem to influence TNF-mediated effects on the virulence of primary rodent malaria infections [18]. Recent studies on naïve volunteers confirmed the contribution of NK cells to total IFNγ production and the existence of memory responses to parasitized erythrocytes [19].

Based on the limited and controversial knowledge on the function of NK cells during the inflammatory process leading to severe malaria and the important role of TNF and IFNγ in the pathogenesis of the complicated disease, the study proposed to correlate the production of pro-inflammatory cytokines by these cells and their subsets, and the clinical outcome in malaria patients in Colombia. The characteristics of NK cells in *P. falciparum*-infected patients of a highly endemic region of Colombia are described. In addition, the degree of NK proliferation and production of IFNγ and TNF production by these cells was explored, with the aim of providing a first glimpse into their profile in naturally-infected populations on this continent.

## Methods

### Subject enrolment

The study took place in Medellin-Colombia, where patients from the northwest region of the country attended the malaria clinic of Grupo Salud y Comunidad, Universidad de Antioquia. A total of 23 acute (non-severe) and seven severe *P. falciparum *malaria patients were recruited. Individuals were included immediately after diagnosis of *P. falciparum *infection was performed by microscopy. Inclusion criteria for this group were: permanent residency in the endemic area, presence of symptomatic malaria and absence of clinical viral infections within 2 months of attendance to the malaria clinic. The degree of clinical impairment was assessed by an experienced medical officer. Patients were classified as having acute malaria or severe malaria according to the WHO criteria[20]. Concomitant infection with other agents was excluded by clinical evaluation and completion of a questionnaire. In addition, 20 healthy controls were included: 10 from Medellin (non-transmission area) and 15 from the Uraba region (malaria-endemic area). Controls were recruited after exclusion of acute disease or infection by physical examination and completion of a questionnaire, which was also used to out rule previous history of malaria. Inclusion criteria for this group were permanent residency in Medellin or the endemic area, absence of symptomatic malaria and any clinically apparent viral infection within 2 months of inclusion. Companions attending the malaria clinics in endemic and non-endemic areas were recruited as controls in a sequential manner for the study.

Volunteers were notified of the risks and their rights and enrolled after providing informed consent. Ethical clearance was granted by the Ethics Committee of the Centro de Investigaciones Medicas, Faculty of Medicine, Universidad de Antioquia (Medellin-Colombia).

### Microscopy for *Plasmodium *detection

Giemsa-Field stained thick/thin blood films were examined with a 100× objective to identify presence of parasites, *Plasmodium *species, parasite density, and schizontaemia. Parasite density was measured by counting the number of asexual parasites per 200 leukocytes, based on a mean count of 8,000 leukocytes per microlitre of blood (theoretical value). A slide was considered negative after examination of at least 300 microscopic fields [21].

### Isolation of peripheral mononuclear cells

Blood samples from volunteers were obtained from peripheral vein and, in the case of malaria subjects, before malaria treatment was administered. Samples of 16 mL were collected into EDTA vacutainer vials from patients and controls and peripheral blood mononuclear cells were separated by density centrifugation (Ficoll-Hypaque 1077). Mononuclear cells were collected from the interface, washed twice by centrifugation with EDTA-PBS at 640 g and 160 g, respectively, for 10 min at 4°C. Cells were then suspended in complete RPMI 1640 medium buffered with 25 mM Hepes and supplemented with 12 mM sodium bicarbonate, 2 mM L-glutamine, 100 U/mL penicillin, 100 mg/mL streptomycin and 5% foetal bovine serum. Cell viability was assessed with 0.1% trypan blue in PBS pH 7.3.

### Flow cytometry

Immunophenotypic analysis of cells was performed using four-colour analysis on a FACSCalibur (Becton Dickinson, San Jose, CA) with CELLQuest Pro software (Becton Dickinson). Cells were stained with the following monoclonal antibodies: fluorescein isothiocyanate (FITC)-conjugated anti-CD56 (SIGMA) and Cychrome (Cy)-anti-CD3 (SIGMA) to determine the percentage of NK cells within the sample. Gating was performed according to size and granulation using forward-scatter versus side-scatter (FSC/SSC) dot plots.

Production of INFγ and TNF by NK cells was determined by flow cytometry. For this, 10^6 ^freshly isolated mononuclear cells per well were incubated with phorbol myristate acetate (PMA) 10 mg/ml plus calcium ionophore 250 ng and brefeldin A 0.4 mg/mL for 6 h at 37°C in 5% CO_2_. Analysis included a description of the total NK cell and subtypes populations according to IFNγ and TNF production in the different groups of study: acute malaria, severe malaria, and healthy volunteers from endemic and non-endemic area. Results were processed using Prisma^®^, and mean values and standard deviations were compared using t-student.

## Results

Natural killer population analysis in the malaria affected group was successfully performed in 17 acute malaria and three severe malaria volunteers. Samples from 10 subjects from Medellin (non-endemic) and 10 from Uraba (endemic region) were processed as controls.

The male/female ratio in the experimental group was 16/4 and the age ranged from 18 to 59 years old (mean 34 ± 14). The male/female ratio in the controls was 13/7 and the age ranged from 18 to 49 years old (mean 33.3 ± 9.9). Among the patients with acute malaria, the most common symptoms were fever (100%), chills (88%) and sweating (47%), while other complaints such as diarrhoea and vomiting were rare. Parasitaemia in these patients ranged between 80 and 9,440 rings/mm^3 ^(mean 5,640 ± 2,281). All patients with severe malaria reported fever, chills, sweating, general malaise, headache, vomiting, two reported dizziness and one had abdominal pain. Parasitaemia in each of these patients was 154,000, 171,000 and 195,000 rings/mm^3 ^(mean 173,500 rings/mm^3^). The diagnosis of severe malaria was based on the high number of vomiting episodes per day and hyperparasitaemia.

### NK cell population distribution

**Figure 1:**
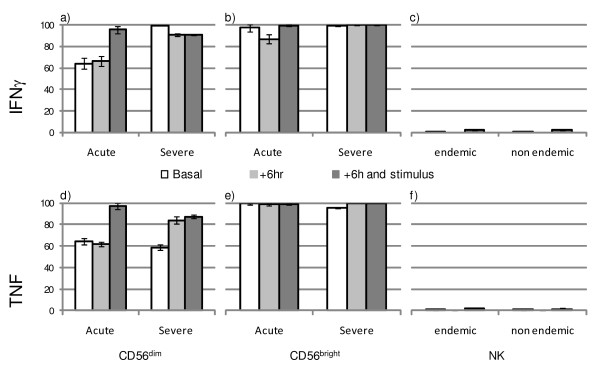
**Proportions of cytokine producing natural killer cells according to clinical status**. Acute malaria *vs*. severe malaria subjects under basal conditions, 6 h after culture (+6 hr) and 6 h after culture plus stimulation with PMA, calcium ionophore Brefeldin A (+6 hr and stimulus). The proportions of IFNγ producing CD56^dim ^(**a**) and CD56^bright ^(**b**) cells, proportions of TNF producing CD56^dim ^(**d**) and CD56^bright ^(**e**) cells. IFNγ (**c**) and TNF (**f**) production by total NK cell population were assessed in healthy controls from endemic and non-endemic regions.

In acute malaria-infected patients, the total CD56^+^/CD3^- ^NK cell population was 9.1 ± 0.3% of which 7.1 ± 0.4% were CD56^dim ^and 2.0 ± 0.2% were CD56^bright^. Meanwhile, in patients with severe malaria, the total population of CD56^+^/CD3^- ^was 19.6 ± 0.6% and the mean CD56^dim ^and CD56^bright ^populations were 16.0 ± 0.3% and 3.6 ± 0.9%, respectively. These proportions of NK cells in acute malaria-infected and severe patients were statistically similar. After PMA and calcium ionophore addition, cells from complicated malaria patients showed higher levels of both CD56^dim ^and CD56^bright ^populations when compared to acute malaria patients (see Figure 1).

The mean CD56^+^/CD3^- ^population in healthy volunteers from a malaria endemic area was 1.2 ± 0.6% and in healthy volunteers from the non-endemic area the mean was 0.7 ± 0.2%. The low numbers of NK cells in these two control groups, made discrimination between CD56^dim ^and CD56^bright ^populations not feasible due to particular ethical constraints of the project to obtain the large volume of blood required to perform such analysis.

### Intracellular IFNγ production

**Figure 2:**
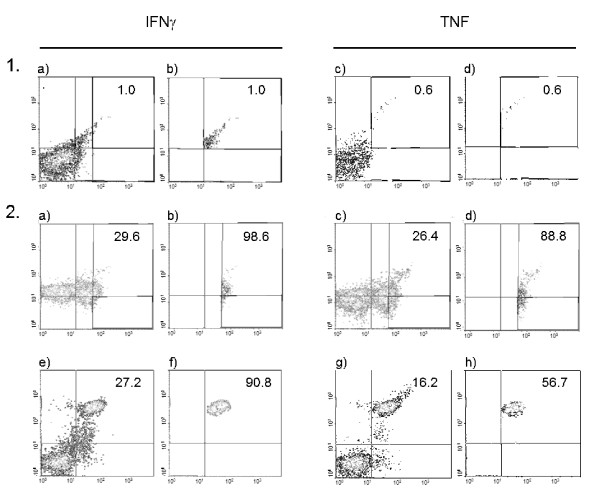
**Representative dot plots demonstrating the method of gating of CD56 cell and subsets and the production of cytokines according to clinical status**. A comparison of non-stimulated total CD56 cells collected from a typical uninfected control (Panels 1 **a**-**d**) and CD56^dim^-CD56^bright ^cells from a malaria infected subject (Panels 2 **a**-**h**), is presented. Numbers in the quadrant represent percent of cytokine cell producing cells (1a, 1c, 2a, 2c, 2e, 2g) and proportion of the cytokine produced by a particular cell subset (1b, 1d, 2b, 2d, 2f, 2h).

NK cells were responsible for 2.4% of the total IFNγ production in subjects with acute malaria, and this proportion was significantly lower compared to the 6.64% observed in severe malaria patients. A mean 76.7 ± 2.3% of NK cells from acute malaria patients produced IFNγ. In patients with severe malaria, 94.6 ± 2.4% of NK cells were IFNγ producers. After stimulus, the proportion of IFNγ-producing NK cells increased to 96.4 ± 3.5% in acute malaria patients and to 99.0 ± 3.6% in severe malaria patients. In controls from endemic and non-endemic areas, baseline proportion of IFNγ producing cells was 0.2 ± 1.1% and increased to 2.5 ± 0.3% after stimulus, in all cases significantly lower than infected subjects. Representative dot plots of these results are presented in Figure 2.

Analysis of basal IFNγ production according to the CD56^dim ^or CD56^bright ^phenotype confirmed that in both acute malaria and in severe malaria subjects, the CD56NK^bright ^population produced significantly higher levels of cytokine (*p *< 0.0001) than the CD56^dim ^population (Figure 1a-b). On the other hand, after 6 hours of stimulus with calcium ionophore, CD56^dim ^cells from malaria-infected patients produced the highest levels of IFNγ regardless of their clinical presentation.

### Intracellular TNF production

The proportion of NK cells producing TNF in acute malaria subjects under basal conditions was 70.3 ± 2.1%. This proportion was significantly lower compared to the 92.5 ± 2.6% observed in severe malaria subjects. The proportion of TNF-producing NK cells rose to > 97% after stimulus in the two groups. Controls from endemic and non-endemic areas exhibited a significant < 0.3% of TNF producing cells, in comparison with infected subjects, either before or after stimulus. Analysis of basal TNF production according to the CD56^dim ^or CD56^bright ^phenotype confirmed that in acute malaria subjects, as well as in severe malaria, the CD56^bright ^population produced significantly higher levels of the cytokine (*p *< 0.0001) than the CD56^dim ^population (Figure 1d-e). However, after 6 hours of stimulus with calcium ionophore, CD56^dim ^cells from acute malaria and from severe malaria subjects produced significantly higher levels of TNF (Figure 1f).

## Discussion

Early events of the immune response against pathogens involve the activation of the innate arm of immunity, which then regulates the subsequent adaptative immune response required to control infection [22]. For many years, NK cells have been proposed to play a significant role in clearance of protozoan parasites [23,24] including blood-stage malaria parasites [25], due to their early activation after infection. Upon contact with infected cells, NK cells proliferate and release cytokines by a complex system of triggering and inhibitory molecules [26,27]. They recognize major histocompatibility complex (MHC) class I molecules via membrane receptors that deliver inhibitory signals of NK-cell cytotoxicity. If target cells lack or express insufficient density of MHC class I molecules, as occurs during infection, cytotoxicity takes place. NK cell-mediated cytotoxicity occurs mainly after binding of NK cells to target cells and release of preformed granules containing perforin and granzymes in the intercellular space, leading to the lysis of target cells within minutes [28].

NK cells are among the first lymphocytes to respond to *P. falciparum*-infected red cells by producing IFNγ as evidenced by *in vitro *[29,30] and *in vivo *studies [31]. Moreover, exposure of naïve volunteers to a single malaria *P. falciparum *infection was able to induce robust and lasting cellular responses of both T and NK cells [19]. Studies on the dynamics of NK activity in malaria patients revealed heterogeneous results, particularly in *P. falciparum *infected individuals. Nevertheless, confirmation of NK activation has been observed in subjects with *Plasmodium vivax *or *P. falciparum *infection [32].

This study detected increased NK cell population in all infected subjects, with similar proportions both CD56^dim ^and CD56^bright ^populations, and, albeit the low number of individuals, this seemed marked in severe malaria cases. Other authors have demonstrated a similar pattern of NK increase in both CD56^dim ^and CD56^bright ^after controlled challenge with *P. falciparum*, reporting an increase of around 12% in NK cells during acute infection [19]. Kassa *et al*. [33] reported normal NK cell counts in *P. falciparum *or *P. vivax *malaria. However, other researchers have reported lower counts of NK cells, and other lymphocytes, in a *Plasmodium berghei *murine model [34] and during acute *P. falciparum *malaria [35-37]. These conflicting results might be due to differences in the baseline values of the absolute counts of the immune cells of the subjects of study. Alternatively, an impact of different geographical locations which has been suggested by other authors, might account for such diverse results [34].

The increased of the NK population proportions during acute and, probably, during severe malaria described in this study, in addition to a positive correlation between NK cytotoxicity and levels of parasitaemia reported by other authors in patients infected with *P. falciparum *[32], confirms the induction of robust cellular responses to *P. falciparum *in naturally infected individuals form the American continent, and highlights the importance of further exploring the role of these cells and theirs subsets in the immunopathology during severe malaria outside Africa.

Activated cells belonging to the two arms of immunity (innate and adaptative) can produce IFN-γ. In malaria, the main cellular sources of the cytokine are T cells, γδ T cells, and NK cells. However, in some subjects, there is little or no evidence of an NK cell response after exposure to *P. falciparum*-infected erythrocytes, whereas in other individuals, NK cells comprise around 70% of IFNγ-producing cells [29,38]. Controversial findings were recently reported on naïve subjects infected with *P. falciparum*, in whom NK cells represented, on average, 14% of IFNγ-producing cells before challenge, rising to 17% immediately after inoculum and by week 20 post-infection, represented only 7%, when T cells reached a peak of production of the cytokine [19]. Such proportions of IFNγ-producing cells are very high compared to the results presented here in all groups of subjects regardless of the presence of infection: 2.43% in acute and 6.64% in severe malaria *vs*. 0.22% in controls, and might reflect the heterogeneity of innate immune responses between donors, and/or populations [30,38] with crucial influencing factors such as type of nutrition [39], among other. Although the effects of the age on these results is possible, the authors consider this unlikely since the groups were homogenous in age and the severe malaria subjects also belonged to the most frequently malaria affected age range in the country. Existence of non-homogenous immune responses is supported by evidence of different strength co-stimulatory signals from myeloid accessory cells and polymorphism among NK regulatory receptors [40,41]. In addition, many reports confirm the requirement of IL-2 as a contributor of IFNγ production by NK cells [19,42]. However, recent reports on pregnant mothers revealed that NK cells are a major source of IFNγ when exposed to *P. falciparum *antigens *in vitro *in the absence of any other co-stimulant. Future *in vivo *studies in populations affected with malaria should explore the significance of IL-2 on the production of IFNγ at early stages of infection and in subjects with different degree of previous exposure to malarial antigens.

TNF is involved in the immune response to malaria as well as in the pathogenesis of severe disease[43,44]. Dysregulation and imbalance of this and other pro-inflamatory cytokines (TNF, IFN-γ, IL-12) versus anti-inflammatory cytokines (IL-4, IL-6, transforming growth factor-1 beta, TGF-1β) appear to be the main mechanisms resulting in the bias towards effective immune actions or tissue damage in the host [45]. The status of TNF in the context of NK cell activation was explored in the current study since these cells have been reported to be early producers of the cytokine after infection. The present results confirmed high levels of TNF-producing NK cells in acute and severe malaria patients, with CD56^bright ^cells exhibiting the highest production of the cytokine in both infected groups.

## Conclusions

The results confirm the important role of not only CD56^bright ^but also of CD56^dim ^NK cell populations as producers of the two cytokines in naturally infected malaria patients in Colombia. Future studies should include exploration of the status of activation of other lymphocyte populations and their relationship with NK cells in infected patients with malaria by *P. vivax *and *P. falciparum *exhibiting different clinical presentations.

## Competing interests

The authors declare that they have no competing interests.

## Authors' contributions

OA and JB designed and performed the NK analysis in blood samples. AV conceived the project and designed the experiments. AM designed the experiments, supervised overall design and development and wrote the manuscript. All authors read and approved the final manuscript.
